# Comment on: Gao B, Lu Q, Wan R, Wang Z, Yang Y, Chen Z, Wang Z. “Monthly versus quarterly fremanezumab for the prevention of migraine: a systemic review and meta-analysis from randomized controlled trials”. *Naunyn Schmiedebergs Arch Pharmacol.* 2021 Apr;394(4):819–828. Epublished November 2020

**DOI:** 10.1007/s00210-021-02156-5

**Published:** 2021-09-28

**Authors:** Steve Barash, Verena Ramirez Campos, Xiaoping Ning, Maurice T. Driessen, Lynda J. Krasenbaum, Karen Carr, Joshua M. Cohen

**Affiliations:** 1 Teva Pharmaceutical Industries Ltd., West Chester, PA USA; 2 Teva Pharmaceutical Industries Ltd., Amsterdam, The Netherlands

**Keywords:** Fremanezumab; Monthly administration; Quarterly administration; Chronic migraine; Episodic migraine; Meta-analysis

## Abstract

Recently, Gao et al. published an article titled “Monthly versus quarterly fremanezumab for the prevention of migraine: a systemic review and meta-analysis from randomized controlled trials” which concluded that monthly administration of fremanezumab led to significant reduction in monthly migraine days (MMD) when compared to quarterly fremanezumab. We have noted a critical flaw in Gao et al. meta-analysis wherein the authors have mistakenly utilized standard error values in place of standard deviation values in performing their pooled analyses. This error directly impacts the study results and conclusions. In this brief communication, we present revised analysis using correct methods. Using the correct SD values, our pooled analysis showed no significant difference in mean change from baseline in MMD between the two fremanezumab dosing regimens (*P* = 0.17). Furthermore, in the corrected subgroup analyses by type of migraine, there were no significant differences in mean change from baseline in MMD between monthly fremanezumab and quarterly fremanezumab (chronic migraine, *P* = 0.50; episodic migraine, *P* = 0.69). Overall, results from our corrected meta-analyses show that there is no significant difference in migraine prevention efficacy between monthly and quarterly fremanezumab dosing.

The recent article “Monthly versus quarterly fremanezumab for the prevention of migraine: a systemic review and meta-analysis from randomized controlled trials” by Bixi Gao and colleagues, published in the April issue of *Naunyn-Schmiedeberg’s Archives of Pharmacology,* concluded that monthly administration of fremanezumab shows better outcomes for preventing migraine headaches than quarterly fremanezumab (Gao et al. 2021). We have noted a critical flaw in this meta-analysis that directly impacts the study results and conclusions. We have also undertaken a revised analysis using correct methods and presented these results.

The Gao et al. analysis pooled published data from three randomized, placebo-controlled, phase 3 studies of fremanezumab administered as a subcutaneous injection monthly or quarterly. The three studies enrolled adult patients with chronic migraine (defined as headache on ≥ 15 days per month and migraine on ≥ 8 days per month) (Silberstein et al. 2017), with episodic migraine (6‒14 headache days with ≥ 4 migraine days per month) (Dodick et al. 2018), and with chronic or episode migraine with failure of 2 to 4 prior migraine preventive medication classes (Ferrari et al. 2019), respectively. In all three studies, the primary outcome was the mean change from baseline in the monthly average number of migraine days (MMD) during the 12-week treatment period.

Gao et al. present forest plots of pooled treatment effects to show the association between fremanezumab dosing regimen (quarterly versus monthly) and the mean change in MMD. For this purpose, the authors used commercial software (Review Manager 5.3) that expects mean values, standard deviations (SDs), and sample sizes as inputs for performing meta-analyses and generating forest plots for continuous outcomes (Review Manager 2014). Their pooled analysis from the three fremanezumab studies showed a mean difference in reduction in MMD in favor of monthly dosing of 0.27 days, with a 95% confidence interval (CI) of 0.11 to 0.42 and a statistically significant *P*-value of 0.0008. The authors mistakenly utilized standard error (SE) values reported in the three fremanezumab publications and used them in place of the SD values required by Review Manager 5.3; SD values are conventionally used for meta-analyses of continuous variables (Deeks et al. 2021). This major flaw led to extremely narrow 95% CIs and thus incorrectly showed significant treatment effects.

Similarly, SE values were used in place of SD values for the subgroup analyses. Subgroup analyses showed that for patients with chronic migraine, there was no significant difference in monthly versus quarterly dosing, while for those with episodic migraine, there was a 0.20-day greater reduction in MMD with monthly versus quarterly dosing (95% CI 0.01, 0.40; *P* = 0.04).

Here we present the results of our corrected meta-analysis using data from the same three fremanezumab clinical trials. Table 1 displays data for the meta-analysis of the overall study population, with mean and SE values taken directly from the study publications and SD values calculated via the formula SD = SE*√N (Higgins et al. 2021). Using the correct SD values, the pooled analysis in the overall population showed no significant difference in mean change from baseline in MMD between the two fremanezumab dosing regimens (*P* = 0.17). Figure 1 shows the incorrect and corrected meta-analyses side-by-side.

**Table 1:** Data for the overall population: mean change in MMD from baseline to week 12

Study	Quarterly dosing				Monthly dosing			
	*N*	Mean	SE	SD	*N*	Mean	SE	SD
Silberstein et al. (2017)	375	− 4.9	0.4	7.7	375	− 5.0	0.4	7.7
Dodick et al. (2018)	288	− 3.4	0.2	3.4	287	− 3.7	0.3	5.1
Ferrari et al. (2019)	276	− 3.7	0.3	5.0	283	− 4.7	0.3	5.0

**Fig. 1:**
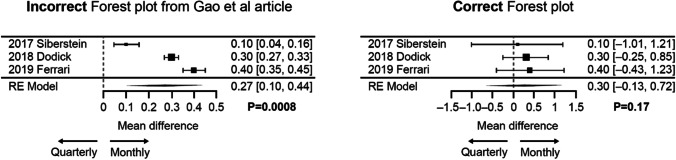
Incorrect and corrected forest plots for mean reductions in MMD from baseline to week 12 with quarterly versus monthly fremanezumab dosing in the overall study population

Data for the subgroup analysis of patients with chronic migraine are displayed in Table 2. The corrected pooled analysis showed no statistically significant difference in mean change from baseline in MMD between the two fremanezumab dosing regimens (*P* = 0.5). Figure 2 shows the incorrect and corrected meta-analyses side-by-side.

**Table 2:** Data for the subgroup analysis in patients with chronic migraine: mean change in MMD from baseline to week 12

Study	Quarterly dosing				Monthly dosing			
	*N*	Mean	SE	SD	*N*	Mean	SE	SD
Silberstein et al. (2017)	375	− 4.9	0.4	7.7	375	− 5.0	0.4	7.7
Ferrari et al. (2019)	169	− 3.9	0.5	6.5	173	− 4.5	0.5	6.6

**Fig. 2:**
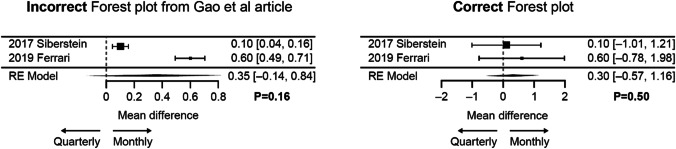
Incorrect and corrected forest plots for mean reductions in MMD from baseline to week 12 with quarterly versus monthly fremanezumab dosing in patients with chronic migraine

Data for the subgroup analysis of patients with episodic migraine are displayed in Table 3. The corrected pooled analysis showed no statistically significant difference in mean change from baseline in MMD between the two fremanezumab dosing regimens (*P* = 0.69). Figure 3 shows the incorrect and corrected meta-analyses side-by-side.

**Table 3:** Data for the subgroup analysis in patients with episodic migraine: mean change in MMD from baseline to week 12

Study	Quarterly dosing				Monthly dosing			
	*N*	Mean	SE	SD	*N*	Mean	SE	SD
Dodick et al. (2018)	288	− 3.4	0.5	8.5	287	− 3.7	0.5	8.5
Ferrari et al. (2019)	107	− 3.7	0.4	4.1	110	− 3.8	0.4	4.2

**Fig. 3:**
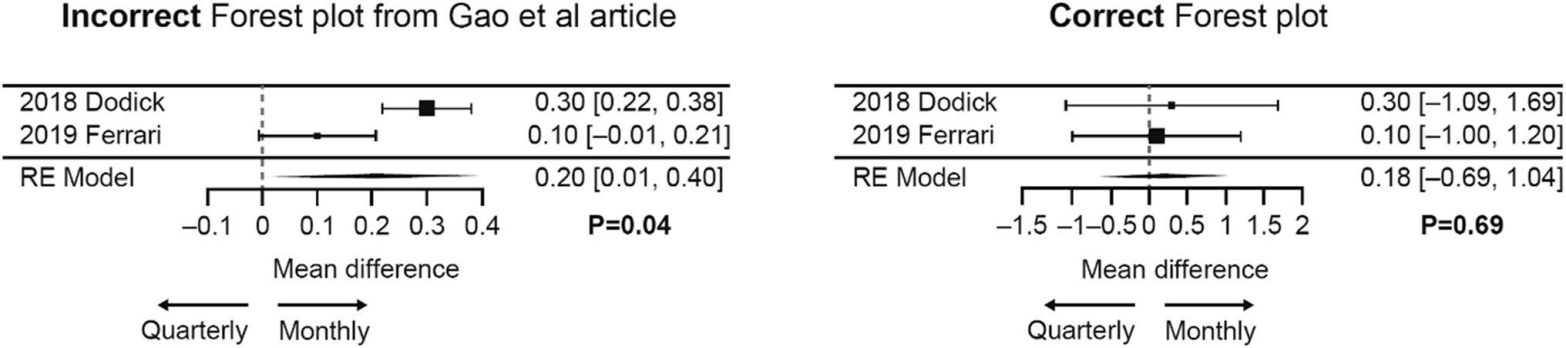
Incorrect and corrected forest plots for mean reductions in MMD from baseline to week 12 with quarterly versus monthly fremanezumab dosing in patients with episodic migraine

To summarize, our corrected meta-analysis using SD values demonstrated no significant differences in efficacy in terms of reduction in MMD between quarterly and monthly dosing of fremanezumab in the overall study population or in the subgroups with chronic or episodic migraine.

It is interesting to note that a previously published meta-analysis by the same authors that pooled data from the three phase 3 studies plus two phase 2b studies found no significant differences in mean change in MMD from baseline to week 12 for monthly versus quarterly fremanezumab dosing (*P* = 0.86) (Gao et al. 2020). However, the authors point out in their more recent publication (Gao et al. 2021) that the results of the prior analysis were based on comparing the two dosage regimens with placebo and did not directly compare the two dosage regimens, hence their rationale for directly comparing quarterly versus monthly dosing in the current analysis. The authors acknowledge that a difference in monthly migraine headache days less than 0.3 days is too small to be of clinical importance, a supposition supported by a lack of a statistically significant difference between quarterly and monthly dosing in the proportion of patients reporting a 50% or greater reduction in monthly migraine headache days (Gao et al. 2021).

In conclusion, our correction to the meta-analysis by Gao and colleagues demonstrates that there is no significant difference in migraine prevention efficacy between quarterly and monthly fremanezumab dosing regimens. The results of our corrected analysis provide evidence further validating that patients with migraine may choose either quarterly or monthly dosing with fremanezumab, depending on convenience and personal preference, without impacting migraine prevention efficacy. We would very much welcome any feedback the authors may have on our interpretation of their study.

## Data Availability

Not applicable.

## Code availability

R software with metafor package, version 2.4
